# Case Report: Delirium and complications resulting from the abuse of compound liquorice tablets

**DOI:** 10.3389/fpsyt.2026.1870259

**Published:** 2026-07-06

**Authors:** Xiwen Tian, Liu Liu, Liyao Huang, Jubo Yin, Yanjuan Zeng, Yao Chen, Shanghao Yang, Xuhui Zhou

**Affiliations:** 1 The School of Clinical Medicine, Hunan University of Chinese Medicine, Changsha, Hunan, China; 2 Department of Addiction Medicine, Hunan Institute of Mental Health, The Second People’s Hospital of Hunan Province (Brain Hospital of Hunan Province), Changsha, Hunan, China; 3 College of Integrated Chinese and Western Medicine, Hunan University of Chinese Medicine, Changsha, Hunan, China

**Keywords:** addiction, compound liquorice tablets, GABA, hypokalemia, opioid withdrawal symptoms, pseudoaldosteronism

## Abstract

**Background:**

Compound liquorice tablets is a cough-suppressing compound formulation containing opium powder and liquorice. With the strict regulation of traditional opioids, this medication has emerged as a novel alternative for substance abuse due to its easy accessibility and low cost; however, its addiction potential and severe adverse complications remain underrecognized and insufficiently addressed in clinical practice.

**Case summary:**

The patient is a 29-year-old male who began self-medicating with compound liquorice tablets for a dry cough after COVID-19 infection. His daily dosage gradually escalated to 200–600 tablets within two years, resulting in established drug dependence. Two days after discontinuing the medication, he developed delirium manifested as confusion, disorientation, visual hallucinations and psychomotor agitation, accompanied by palpitations, hypertension, tremors, rhinorrhea, vomiting, severe hypokalemia, and bilateral lower limb edema. Organic brain diseases were excluded by systematic examinations. According to the ICD-10 diagnostic criteria, he was diagnosed with opioid-induced mental and behavioral disorders, hypokalemia and hypertension. We implemented a benzodiazepine tapering regimen to control delirium and sympathetic excitation associated with withdrawal symptoms, combined with antidepressants and antipsychotics to improve mood, anxiety, and psychotic symptoms, Under the guidance of a cardiologist, we actively managing hypertension and correcting internal environmental disturbances. Following comprehensive management, the patient’s withdrawal symptoms and delirium resolved, with stable emotional state and blood pressure, resulting in successful withdrawal.

**Conclusion:**

This is the first reported case of delirium caused by withdrawal from compound liquorice tablets. It provides preliminary insights for clinical identification, diagnosis, and multidisciplinary management of dependence on compound liquorice tablets, as well as related withdrawal symptoms and complications. This case also alerts to the emerging risk of abuse associated with new substances such as compound liquorice tablets and underscores the need for stricter prescription controls and patient education regarding the risks of abuse.

## 1 Introduction

Compound liquorice tablets is a compound preparation containing traditional Chinese medicinal ingredients (1). Each tablet contains 4 mg of opioid powder or poppy fruit extract powder, 112.5 mg of liquorice extract powder, 2 mg of camphor, 2 mg of star anise oil, and 2 mg of sodium benzoate. Clinically, it is primarily indicated for treating coughs associated with upper respiratory tract infections, bronchitis, and the common cold. During the COVID-19 pandemic, this medication was recommended by expert consensus for the symptomatic treatment of productive and dry coughs, serving as a home medicine cabinet staple (2). Furthermore, glycyrrhizic acid and its related formulations have demonstrated favorable efficacy in shortening the ICU stay of critically ill COVID-19 patients (3). Although this medication is widely used clinically and easily accessible to the public, there is a general lack of awareness regarding the risk of addiction associated with its opioid components.

Stringent legal and clinical regulation of traditional drugs such as heroin and medical opioids has driven substance users to seek new alternatives (4). Some herbal compound preparations serve as a perfect cover for opioid abuse (5). In China, the abuse of compound liquorice tablets is on the rise (6, 7).However, the risks of addiction and adverse consequences resulting from abuse have not been fully recognized or systematically studied, and relevant clinical experience remains limited. we report a rare case of severe withdrawal delirium and multiple complications secondary to compound liquorice tablets abuse. Drawing on comprehensive management experience, it provides a basis for the diagnosis and treatment of compound liquorice tablets dependence and serves as a warning to the public and clinical practitioners regarding this new potential form of opioid abuse. This case report was written in accordance with the CARE guidelines to standardize clinical data presentation.

## 2 Case presentation

### 2.1 History of present illness

A 29-year-old unemployed man presented to the stroke emergency department on November 13, 2024, with altered mental status, disorientation, palpitations, tremors, severe vomiting, and muttering to himself. Following an initial assessment of vital signs, a neurological examination, and a head CT scan, acute intracranial hemorrhage or extensive cerebral infarction were ruled out. Based on his clinical presentation and preliminary medical history, he was provisionally diagnosed with “substance use disorder” and admitted to the Addiction Medicine Center for inpatient treatment. Upon admission, a detailed medical history was taken, revealing that he had a history of using compound liquorice tablets for nearly two years.

Shortly after China implemented the “Category B Management” policy for COVID-19, the patient contracted COVID-19 in January 2023. Following treatment during the acute phase, he was left with a persistent dry cough and irritability. Based on previous self-medication experience, he initiated oral compound liquorice tablets for cough relief. Initially, he took 9 tablets daily, but his dry cough did not improve significantly, he gradually increased his daily dose. By the third day, he was taking 18 tablets daily, which provided slight relief from his cough. He reported feeling happy and relaxed and continued to gradually increase his daily dose. After three weeks of treatment, his daily intake reached 100 tablets (one bottle). At this point, his persistent cough had subsided, and he planned to discontinue the medication. On the day he stopped taking the tablets, he experienced generalized weakness, yawning, a runny nose, and dry mouth, accompanied by palpitations, restlessness, and emotional instability. He believed these were symptoms of COVID-19 that had not been fully controlled. After resuming the compound liquorice tablets, the aforementioned symptoms were largely alleviated, and he reported experiencing brief feelings of euphoria and relaxation. In March 2023, the patient was taking approximately 200 compound liquorice tablets daily. He noticed that the effects were gradually diminishing and continued to raise the dosage.

Since April 2023, the patient has maintained a daily dose of 200–600 compound liquorice tablets, but most of the time he took 500 tablets daily (equivalent to 2000 mg of opioid powder or poppy fruit extract powder, 56250 mg of liquorice extract powder, and 1000 mg of camphor). He reported that taking the entire dose at once provided the greatest sense of comfort, and since a bottle costs only cost 12 RMB that reinforced compulsive drug-taking behavior, he frequently purchased large quantities of the medication. This drew the attention of staff at a pharmacy near his home, who refused to sell it to him. The patient then began seeking out intermediaries to purchase the medication. The patient recognized that his use of compound liquorice tablets had far exceeded his expectations. Whenever he attempted dose reduction or discontinuation, he experienced severe palpitations, mild tremors, profuse sweating, a runny nose, myalgia, arthralgia, formication, nausea, emotional tension, irritability, restlessness and severe insomnia. All above symptoms were relieved upon resuming the compound liquorice tablets. Due to intolerable withdrawal distress, he never achieved successful abstinence.

On November 12, 2024, the patient went on a trip with his partner. To prevent his partner from discovering that he was taking medication, he voluntarily stopped taking compound liquorice tablets. On the first day after discontinuing the medication, he experienced symptoms such as anxiety, palpitations, and restlessness. On November 13, 2024 (the second day after discontinuation), the aforementioned symptoms progressively worsened, and he developed profuse sweating, goosebumps, muscle aches, frequent yawning, and a runny nose. Subsequently, the patient’s generalized weakness significantly worsened, causing him to fall to the ground. This was followed by behavioral disturbances; he reported seeing a group of people rushing toward him but found it difficult to move his legs. He felt extreme fear and despair and gradually began to speak incoherently, asking his partner, “Who are you?” and remaining wary of her. He could not recall the rest of the events. This occurred on the second day of drug withdrawal. Upon emergency admission, he presented with confused consciousness. Vital signs on initial assessment were as follows: body temperature 36.3 °C, respiratory rate 20 breaths per minute, pulse 110 beats per minute, and blood pressure 166/98 mmHg.

### 2.2 Personal and past medical history

The patient had a 2-year smoking history of 10 cigarettes per day and occasionally used heroin nine years ago. He denies using any other psychoactive substances concurrently with his abuse of compound liquorice tablets. During the period of taking compound liquorice tablets, the patient frequently experienced weakness in the lower limbs. In July 2023, the patient sought medical attention for bilateral lower limb edema. Comprehensive testing revealed only hypokalemia, and the patient was diagnosed with “hypokalemia and hypertension” but did not receive standard antihypertensive treatment. Since then, the patient has experienced bilateral lower limb edema 1–3 times per month. While taking compound liquorice tablets, the patient occasionally experienced dizziness and generalized weakness. The patient had several brief episodes of “syncope” lasting from a few seconds to 3 minutes, which resolved spontaneously. During these episodes, there were no limb convulsions, no tongue biting, and no urinary or fecal incontinence; the specific diagnosis remains unclear.

### 2.3 Further diagnostic workup

#### 2.3.1 Physical and psychiatric examination

Further physical examination revealed a body weight of 93 kg, cold and clammy skin, sweating on the forehead, gooseflesh-like skin changes, tremors in the muscles of both upper limbs, pitting edema around both ankles, and an inability to cooperate during muscle strength testing of both lower limbs.

Psychiatric assessment: General condition: Passive and uncooperative during conversation; Consciousness and orientation: Impaired clarity of consciousness; impaired orientation to time and person; Perception and attention: visual hallucinations present; inability to concentrate; Affect: marked anxiety, fear, irritability, and psychomotor agitation; Cognition and insight: unable to cooperate with cognitive function tests; lack of insight; Basic drives: decreased appetite, sleep disturbance; Associated physical symptoms: diarrhea.

#### 2.3.2 Psychometric scales

The patient recently underwent a comprehensive assessment using standardized scales. The total score of the Chinese version of the Hamilton Anxiety Rating Scale (HAMA) was 46, indicating extremely severe anxiety symptoms. The score on the Positive and Negative Syndrome Scale (PANSS) was 97, reflecting relatively severe psychotic symptoms. The Clinical Opiate Withdrawal Scale (COWS) score was 26, suggesting moderate withdrawal symptoms. The Visual Analog Scale (VAS) score was 10, indicating that the patient has a strong craving for compound liquorice tablets.

#### 2.3.3 Laboratory and imaging findings

Laboratory tests showed normal levels of the three inflammatory markers, lactate, blood glucose, blood ammonia, liver function, kidney function, cardiac enzymes, myoglobin, troponin, NT-proBNP, and plasma D-dimer; normal results for the five thyroid function tests, stool routine, and urinalysis; and a complete blood count revealing a mild elevation in white blood cell count (10.51 × 10^9^/L), with an elevated neutrophil count (7.18 × 10^9^/L). Homocysteine is elevated (19.4 μmol/L); coagulation panel shows a shortened prothrombin time (10.90 seconds); electrolyte panel shows a decreased serum potassium concentration (3.14 mmol/L). Urine drug screening shows a positive result for morphine.

Cerebral MRI and SWI showed no significant abnormalities; head, chest, abdominal, and pelvic CT scans (both routine and 3D) showed no significant abnormalities; EEG revealed mild background abnormalities with poor regulation and amplitude, increased β-wave activity, and no epileptiform discharges. Electrocardiography demonstrated sinus rhythm with T-wave flattening in leads V4–V6. Echocardiography presented left atrial enlargement and interventricular septal thickening.

### 2.4 Diagnosis

#### 2.4.1 Differential diagnosis

Based on a comprehensive analysis of the onset pattern and functional status, the patient suffered from acute-onset altered consciousness, emotional lability and psychotic symptoms without trauma or high fever. Emergency CT, MRI, EEG, physical examination, and neurological examination did not suggest organic conditions such as stroke. The patient developed delirium two days after discontinuing the medication, which is inconsistent with the characteristics of acute camphor poisoning or poisoning from other adulterants. However, due to objective constraints, it was not possible to conduct an analysis of the tablet’s composition or secondary toxicological testing of bodily fluids; this constitutes a limitation of this case report. The patient denied a history of using other opioid medications such as heroin, morphine, or oxycodone; therefore, a positive urine morphine test due to exogenous opioids and psychiatric disorders caused by other psychoactive substances can be preliminarily ruled out. No obvious psychological stressors or persistent depressive mood were identified, enabling the temporary exclusion of severe stress reaction, adjustment disorder and depressive disorder.

#### 2.4.2 Addiction-related diagnostic basis

Based on the patient’s clinical presentation, five key diagnostic criteria have been met over the past year (1): a strong craving and urge to use compound liquorice tablets (2); an inability to control the frequency or dosage of compound liquorice tablets (3); tolerance, requiring higher doses to achieve the same effect; Withdrawal symptoms upon dose reduction or discontinuation, which are relieved by resuming medication (5); Gradually increasing time and energy spent obtaining compound liquorice tablets, accompanied by a loss of interest in other activities (6); Continued use of compound liquorice tablets despite adverse consequences, such as deteriorating health, emotional and social problems resulting from long-term use, and significant impairment in daily life and social functioning, including exacerbated family conflicts and inability to maintain employment. Based on these criteria and the ICD-10 guidelines (8), the patient was diagnosed with “F11.2 Opioid dependence syndrome”. After discontinuing the medication, the patient exhibited delirium, meeting the criteria for “F11.4 Opioid withdrawal state with delirium”. All of the above diagnoses fall under “F11 Mental and behavioral disorders due to use of opioid”. Based on laboratory tests, blood pressure monitoring results, and the patient’s medical history, the patient can be diagnosed with “hypokalemia, hyperhomocysteinemia, and hypertension.”.

### 2.5 Treatment

**Figure 1:**
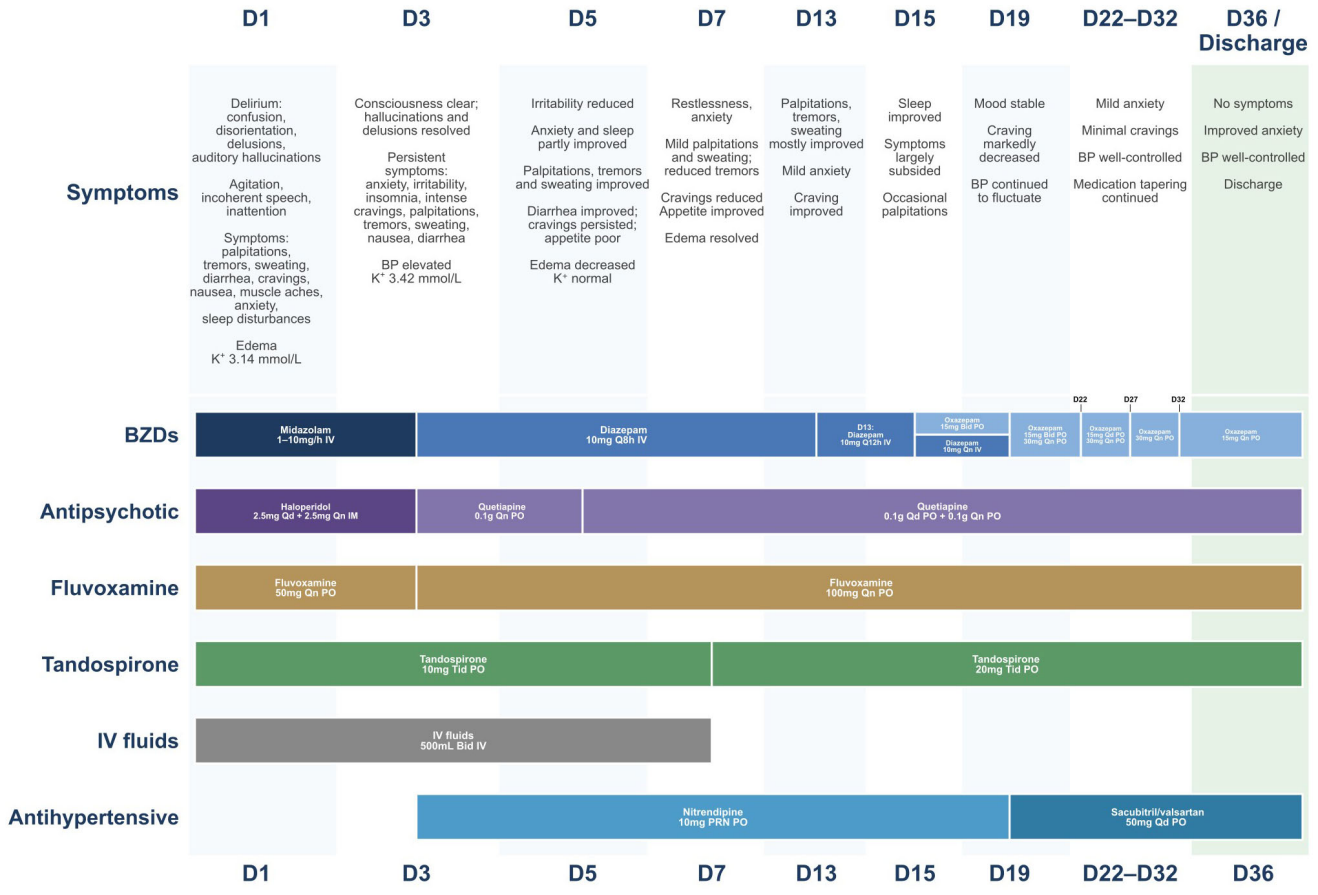
Timeline of clinical manifestations and medication administration during hospitalisation. BZDs, benzodiazepines; IV, intravenous; PO, oral; Qd, once daily; Q12h, every 12 hours; Qn, once nightly; Tid, three times daily; Bid, twice daily; PRN, as required; BP, blood pressure; K^+^, serum potassium; D, days of hospitalisation.

Detailed medication administration is provided in **Figure 1**. Upon admission, a comprehensive, symptom-focused treatment plan was initiated. For withdrawal-induced delirium, continuous intravenous midazolam infusion (1–10 mg/h, adjusted as needed) was administered under close vital sign monitoring. Oral tandospirone citrate (10 mg, three times daily) and fluvoxamine maleate (50 mg, once nightly) were prescribed for anxiety management. Intramuscular haloperidol (2.5 mg, once daily and once nightly) was used to control psychotic symptoms and psychomotor agitation. Given poor oral intake and frequent diarrhea, daily intravenous fluid replacement with 1000 mL solution containing 3 g potassium was administered to correct electrolyte imbalance, which was tapered and discontinued based on subsequent serum potassium levels and dietary recovery.

On day 3, the patient regained clear consciousness with complete resolution of hallucinations and delusions. However, significant withdrawal symptoms including palpitations, hand tremors, sweating, nausea, diarrhea and muscle pain persisted, accompanied by continued anxiety, irritability, and insomnia, as well as a diminished sense of interest. He exhibited a strong craving for compound liquorice tablets. His psychiatric scale scores showed improvement (COWS 22, HAMA 41, PANSS 67), and a craving score (VAS 10). Midazolam was discontinued, and diazepam (10 mg, every 8 hours) was initiated for sustained withdrawal control. Haloperidol was switched to oral quetiapine fumarate (0.1 g, once nightly) to improve sleep and control residual psychotic symptoms. The nightly dose of fluvoxamine maleate was increased to 100 mg for enhanced anxiolytic efficacy. During treatment, the patient’s blood pressure was found to be elevated, so nitrendipine tablets were temporarily administered orally.

On Day 5, the patient’s irritability had decreased compared to before, and symptoms of palpitations, hand tremors, and sweating had slightly improved. Edema had subsided, but drug craving and anxiety persisted with frequent nighttime awakening. The quetiapine dose was increased to 0.1 g every 12 hours to improve sleep and stabilize mood.

On day 7, the patient still presented with restlessness and anxiety, while craving decreased, appetite improved, serum potassium returned to normal range, and edema completely resolved. The daily dosage of tandospirone citrate was increased to 60 mg, and intravenous fluid and potassium supplementation were discontinued. Further psychometric improvements were noted: COWS 13, VAS 7, HAMA 27, PANSS 50.

On day 13, most physical withdrawal symptoms were well-controlled, with improved scores (COWS = 6, VAS = 5, HAMA = 19, PANSS = 41). The diazepam dose was adjusted to 10 mg every 12 hours.

On day 15, sleep quality recovered markedly and physical withdrawal symptoms nearly resolved. Daytime diazepam was discontinued and replaced with oral oxazepam (15 mg, twice daily).

On day 19, emotional status remained stable with significant decrease of craving and optimal withdrawal control. Nighttime diazepam was discontinued, and oxazepam (30 mg nightly) was introduced. Due to recurrent blood pressure fluctuations, sacubitril/valsartan (50 mg, once daily) was initiated on the recommendation of a cardiologist for blood pressure control and to prevent ventricular remodeling. His COWS, VAS, and HAMA scores were 4, 1, and 10, respectively.

During the subsequent hospitalization period, oxazepam tablets were gradually tapered and discontinued. The patient received weekly cognitive behavioral therapy (CBT) and relapse prevention counseling throughout the inpatient stay. He was discharged in December 2024 with minimal residual anxiety and well-regulated blood pressure, (COWS 3, HAMA 9). Maintenance medications at discharge included quetiapine fumarate (200 mg daily), tandospirone citrate (60 mg daily), sacubitril/valsartan (50 mg daily), and fluvoxamine maleate (100 mg once nightly).

### 2.6 Outcome and follow-up

During telephone follow-up with the patient, it was noted that the patient attended follow-up appointments regularly and adhered to the prescribed medication regimen during the first two months after discharge. Blood pressure remained stable with sacubitril/valsartan therapy, and there were no episodes of edema or syncope. During the third-month follow-up, the patient reported taking Qiangli Pipa Syrup (an oral medication containing poppy shell extract); however, the patient refused to provide further details. Consequently, it was impossible to determine whether this represented a relapse, a new addiction, or a mere coincidence. This constitutes one of the limitations of this study, and we recommended that the patient seek consultation at the addiction clinic.

## 3 Discussion

### 3.1 Abuse risk and clinical diagnostic recognition of compound liquorice tablets

Compound liquorice tablets is a compound preparation containing opium powder. Although the opium content per tablet is low, this ingredient poses the core risk of abuse associated with the product. With increasingly stringent regulation of traditional opioids such as heroin, this medication has become a new target for abuse among substance-dependent individuals due to its easy availability and low cost (9). Although the package insert clearly warns of addiction potential and recommends a treatment course of no more than 7 days, the general public lacks adequate awareness of its addictive properties. Furthermore, compound preparations containing herbal ingredients are often perceived as completely safe, which readily leads to excessive dosage and prolonged medication duration, ultimately progressing to adverse consequences.

In the present case, the patient admitted during the medical history review that he initially took compound liquorice tablets solely to relieve persistent post-COVID-19 dry cough, with no awareness of its potential dependence risk. To achieve a “euphoric sensation,” he rapidly increased the dosage, which served as positive reinforcement for the progression of his dependence on compound liquorice tablets. When he stopped taking the medication, the immediate onset of a series of withdrawal symptoms compelled persistent medication intake, representing a typical negative reinforcement mechanism in the development of substance dependence. The patient eventually progressed to severe compound liquorice tablets dependence. The opioid powder in compound liquorice tablets is the primary factor contributing to the risk of addiction and the occurrence of withdrawal symptoms. Liquorice extract and camphor are the main substances responsible for systemic metabolic complications and metabolic toxicity (10). In this case, the patient’s acute delirium and multiple metabolic complications at admission complicated the initial diagnosis.

### 3.2 Neural mechanisms underlying withdrawal symptoms and delirium induced by compound liquorice tablets dependence

Abrupt discontinuation or rapid dose tapering after long-term ultra-high-dose administration of compound liquorice tablets containing opium powder disrupts the adaptive homeostasis of the central nervous system, triggering a complex neuropsychiatric syndrome. The occurrence of physical withdrawal symptoms and withdrawal delirium is primarily attributed to severe dysregulation of central neurotransmitter systems, particularly functional collapse of the locus coeruleus (LC) noradrenergic system and the ventral tegmental area (VTA) dopamine system. On the one hand, chronic opioid exposure compensatorily upregulates the cAMP/PKA/CREB signaling pathway in the locus coeruleus (11–13). Upon sudden drug cessation, the opioid-mediated inhibitory tone is abruptly abolished, rendering LC-noradrenergic neurons in a state of disinhibition with sharply elevated firing frequency. This further drives tyrosine hydroxylase (TH)-mediated excessive norepinephrine synthesis. The resultant intense sympathetic hyperactivity and autonomic storm not only directly induce somatic manifestations such as palpitations, diaphoresis, and tremors, but also lay the pathophysiological foundation for central hyperarousal and delirium. On the other hand, prolonged drug exposure alters the intrinsic synaptic plasticity of dopaminergic neurons and attenuates GABAergic inhibitory function (14–17). During withdrawal, rebound overactivation of the VTA dopaminergic system leads to excessive dopamine release (18). Dopaminergic dysregulation within the mesolimbic pathway directly contributes to severe psychotic delirium features, including irritability, disorientation, auditory hallucinations, and paranoid delusions. Chronic opioid exposure also modulates homeostatic regulation of cholinergic tone and induces adaptive changes in glutamatergic and GABAergic pathways (19, 20). While these adaptive mechanisms tend to stabilize during sustained medication use, they render neural circuits vulnerable to acute perturbation upon drug discontinuation. Furthermore, patients frequently experience hypokalemia during substance abuse; while this does not independently induce delirium, it can lower the central nervous system’s delirium threshold. We must consider that electrolyte disturbances, primarily hypokalemia, during the withdrawal period from compound liquorice tablets may act as a contributing factor to delirium.

### 3.3 Management of withdrawal symptoms and delirium related to compound liquorice tablets dependence

Opioid withdrawal-induced delirium is a rare but life-threatening critical psychiatric emergency, which has only been documented in a limited number of case reports (21–23). The core therapeutic priorities include rapid stabilization of vital signs, control of acute behavioral and psychotic symptoms, and correction of internal environmental disturbances.

For conventional opioid withdrawal, classic opioid receptor agonists or partial agonists (such as buprenorphine and methadone) and benzodiazepines (diazepam, lorazepam) are commonly adopted for tapering substitution therapy (24–27). However, in the present case complicated by marked sympathetic overactivation concurrent with acute delirium, previous case reports have indicated that low-dose buprenorphine initiation under opioid withdrawal background may precipitate or exacerbate delirium (28, 29). We do not routinely use methadone. In this case, the patient had concomitant hypokalemia and was at risk of QTc prolongation; methadone could further prolong the QTc interval and increase the risk of malignant arrhythmias, making it a relative contraindication (30). Therefore, opioid substitution therapy was avoided in this patient. Instead, targeting the core pathophysiological mechanisms—excessive activation of the locus coeruleus noradrenergic neurons and subsequent autonomic storm and central hyperarousal—a sequential tapering regimen of benzodiazepines including midazolam, diazepam, and oxazepam was implemented. As positive allosteric modulators of GABA-A receptors, benzodiazepines potently enhance central GABAergic inhibitory tone (31, 32). By modulating GABA interneuron pools within the locus coeruleus, they effectively suppress overexcitation of noradrenergic (NE) neurons in the locus coeruleus, thereby alleviating withdrawal symptoms such as anxiety, agitation, and autonomic dysfunction. Upon admission, the patient presented with severe withdrawal delirium, agitation, and an extremely high risk of impulsivity, requiring rapid sedation to control symptoms. Midazolam, with its rapid onset, short half-life, and rapid metabolism, allows physicians to flexibly adjust the dosage based on the patient’s level of consciousness and degree of agitation, thereby achieving rapid and precise sedation (33). Throughout the treatment period, 24-hour monitoring of ECG, respiration, blood pressure, and consciousness is critical for medication safety. Once delirium and agitation were significantly controlled, the patient was switched to the long-acting benzodiazepine diazepam to ensure a smooth transition and avoid fluctuations in sedation and withdrawal rebound caused by frequent administration of short-acting medications. Subsequently, the regimen is gradually switched to oral oxazepam, as its metabolism does not rely on hepatic CYP450 enzymes (34), resulting in better tolerability and facilitating gradual dose reduction and smooth discontinuation.

Meanwhile, dopamine receptor antagonists were combined to precisely antagonize mesolimbic dopaminergic hyperactivity for the management of hallucinations, delusions, and other psychotic symptoms during withdrawal (35, 36). Although the use of haloperidol carries a potential risk of QTc interval prolongation (37), we decided to administer a short-term, low-dose course of haloperidol after fully informing the patient’s family and weighing the risks and benefits. In terms of rapidly controlling acute agitation and reducing the risk of impulsivity, this approach may offer greater benefits than other oral medications. Another important point is that during treatment with midazolam and haloperidol, we dynamically monitored changes in the patient’s mental status and oxygen saturation and implemented 24-hour continuous ECG monitoring, with the QTc interval as a key indicator of concern. This rigorous monitoring was a necessary measure to ensure safety, and the patient did not exhibit any abnormalities in the QTc interval during medication. Of course, in settings with limited monitoring capabilities, this aggressive treatment regimen should be approached with particular caution. Studies have shown that quetiapine can serve as an adjunctive medication during opioid withdrawal (38), particularly in alleviating physical pain, reducing anxiety, and improving sleep. Recent studies increasingly support the use of antidepressants to improve mood during withdrawal (39, 40), In this case, serotonin reuptake inhibitors such as fluvoxamine are used to modulate serotonin levels and alleviate patients’ anxiety. Tandospirone, a selective 5-HT1A receptor partial agonist, is increasingly being used in combination to enhance anxiolytic effects (41); however, the risks and benefits must also be evaluated when multiple medications are combined. Vital signs were closely monitored throughout hospitalization, with fluid resuscitation and correction of electrolyte imbalances. This integrated strategy combining supportive care and targeted receptor modulation successfully blocked the core pathological pathway of withdrawal delirium, enabling safe and smooth withdrawal. Additionally, studies have also shown that α_2_-adrenergic receptor agonists (such as clonidine) are important medications for opioid withdrawal treatment (42, 43). Clonidine can help alleviate symptoms of sympathetic hyperactivity caused by opioid withdrawal and synergistically regulate blood pressure, making it theoretically suitable for this patient; however, due to limitations in drug availability, it was not actually administered, which is one of the limitations of this case study.

### 3.4 Complications associated with compound liquorice tablets misuse

Beyond opioid dependence and withdrawal syndrome, another life-threatening risk associated with the abuse of compound liquorice tablets stems from severe metabolic and endocrine disorders caused by their liquorice content (44). Glycyrrhizin, the primary active component of liquorice extract, has been approved by the U.S. Food and Drug Administration as a food additive and is widely applied in modern clinical practice for its anti-inflammatory and hepatoprotective properties (45–47). Nevertheless, long-term intake at extremely high doses may lead to severe endocrine and metabolic disorders.

In this case, the patient experienced persistent lower extremity edema, hypokalemia, and hypertension following the use of compound liquorice tablets. After discontinuing the medication, the edema resolved completely. Although the lack of plasma renin and aldosterone level tests constitutes a limitation in the diagnosis of this case, the Naranjo Adverse Drug Reaction Probability Scale score was 9 (definite causal association), and after ruling out liver or kidney disease, lower extremity venous thrombosis, and diuretic abuse, the clinical presentation is highly consistent with pseudoaldosteronism (PsA) induced by compound liquorice tablets (48). This condition is predominantly driven by metabolic toxicity of glycyrrhizin and its metabolite glycyrrhetinic acid. Structurally similar to aldosterone, glycyrrhetinic acid directly binds to mineralocorticoid receptors and mimics aldosterone bioactivity. Furthermore, it inhibits the activity of renal 11β-hydroxysteroid dehydrogenase type 2 (11-HSD2), an enzyme responsible for converting cortisol into inactive cortisone, leading to substantial cortisol accumulation. Both mechanisms result in excessive activation of renal aldosterone receptors, followed by pathological water-sodium retention and enhanced potassium excretion (49–51). The aforementioned mechanisms trigger various disorders and pathological states, with the most direct effects being hypokalemia, metabolic alkalosis, edema, and hypertension. Such severe endocrine and metabolic disturbances can exacerbate central nervous system dysfunction and autonomic imbalance, potentially playing a significant role in promoting the onset and progression of acute withdrawal delirium.

Current research indicates that the daily threshold dose of glycyrrhizic acid causing hypokalemia is 101mg (52), and the patient’s actual intake far exceeded this cutoff, confirming overt overconsumption. Mild hypokalemia and related mild complications usually resolve spontaneously after discontinuation of liquorice-containing preparations. However, numerous studies have documented that chronic excessive intake of liquorice or high-glycyrrhizin formulations may precipitate catastrophic adverse events including severe hypokalemia, fatal arrhythmia, refractory hypertension, muscle paralysis, rhabdomyolysis, coma, and even myocardial infarction, with some complications posing life-threatening risks (53–55).

It is worth noting that in this case, the patient was diagnosed with hypertension after long-term use of compound liquorice tablets. Although acute water-salt metabolism imbalance was corrected through withdrawal therapy and potassium supplementation, the patient’s blood pressure remained poorly controlled. Furthermore, echocardiography revealed thickening of the interventricular septum, and electrocardiography showed T-wave changes, suggesting that the long-term metabolism of excessive compound liquorice tablets in the body has caused irreversible structural remodeling of the cardiovascular system.

In addition, the recurrent brief “syncope” episodes experienced by the patient during long-term medication use may also be related to metabolic disturbances. In this case, the patient’s last dose consisted of 500 tablets of compound liquorice tablets (containing 1000 mg of camphor). The neurotoxic dose of camphor is >50 mg/kg body weight (56); given the patient’s weight of 93 kg, the toxic dose would be 4,650 mg or more. Studies have indicated that camphor poisoning takes effect very rapidly (5–15 minutes), primarily affecting the central nervous system (57). Symptoms often include confusion, tremors, delirium, or seizures. Most individuals no longer exhibit symptoms beyond a 24–48 hour window; however, in certain cases, physiological disturbances may persist for a longer duration (58). Although the patient in this case did not experience acute camphor poisoning symptoms, the metabolic toxicity of camphor may have been a contributing factor to the patient’s previous episodes of “syncope” and the current episode of delirium.

## 4 Conclusion

Withdrawal delirium caused by the abuse of compound liquorice tablets is a rare and critically serious clinical condition resulting from the interplay of multiple factors. Its core pathophysiological mechanism involves excessive excitation of the locus coeruleus-sympathetic nervous system induced by opioid withdrawal. Furthermore, the secondary pseudoaldosteronism caused by glycyrrhizic acid metabolism and the associated internal environmental disturbances may have a synergistic exacerbating effect, while camphor and other potential adulterants may also promote disease progression. When managing patients presenting with both acute psychiatric disorders and complex physical complications, clinicians should maintain a high level of diagnostic sensitivity. Regarding treatment, a comprehensive management strategy centered on sequential tapering of benzodiazepines, combined with antipsychotics and antidepressants, while actively correcting electrolyte imbalances and managing cardiovascular complications, has proven safe and effective in clinical interventions for compound liquorice tablets withdrawal. For patients dependent on compound liquorice tablets, the α_2_-receptor agonist clonidine may serve as a potential adjunctive medication to optimize the management of withdrawal symptoms and blood pressure control. This case report is the first to document delirium associated with withdrawal from compound liquorice tablets. It provides a reference for the clinical identification, individualized treatment, and development of prevention and control strategies for this condition. It also serves as a warning that multi-stakeholder collaboration is needed to strengthen prescription control, risk education, and drug regulation to reduce the harms associated with the misuse of such potential opioid-containing combination formulations.

## Data Availability

The original contributions presented in the study are included in the article/supplementary material, further inquiries can be directed to the corresponding author.
